# *TP53* mutated glioblastoma stem-like cell cultures are sensitive to dual mTORC1/2 inhibition while resistance in *TP53* wild type cultures can be overcome by combined inhibition of mTORC1/2 and Bcl-2

**DOI:** 10.18632/oncotarget.11205

**Published:** 2016-08-11

**Authors:** Subramanian Venkatesan, Marlous Hoogstraat, Ester Caljouw, Tessa Pierson, Jochem K.H. Spoor, Lona Zeneyedpour, Hendrikus J. Dubbink, Lennard J. Dekker, Mariëlle van der Kaaij, Jenneke Kloezeman, Lotte M.E. Berghauser Pont, Nicolle J.M. Besselink, Theo M. Luider, Jos Joore, John W. Martens, Martine L.M. Lamfers, Stefan Sleijfer, Sieger Leenstra

**Affiliations:** ^1^ Department of Neurosurgery, Brain Tumor Center Erasmus MC, Rotterdam, The Netherlands; ^2^ Department of Medical Oncology, University Medical Center Utrecht, Utrecht, The Netherlands; ^3^ Center for Personalized Cancer Treatment (CPCT), University Medical Center Utrecht, Utrecht, The Netherlands; ^4^ Pepscope BV, Utrecht, The Netherlands; ^5^ Department of Neurology, Erasmus MC Cancer Institute, Rotterdam, The Netherlands; ^6^ Department of Pathology, Erasmus MC Cancer Institute, Rotterdam, The Netherlands; ^7^ Department of Genetics, University Medical Center Utrecht, Utrecht, The Netherlands; ^8^ Department of Medical Oncology, Erasmus MC Cancer Institute, Rotterdam, The Netherlands; ^9^ Cancer Genomics Netherlands, Erasmus MC Cancer Institute, Rotterdam, The Netherlands; ^10^ Department of Neurosurgery, St. Elisabeth Hospital Tilburg, Tilburg, The Netherlands

**Keywords:** brain tumor, personalized medicine, genetic biomarkers, small molecule kinase inhibitors, resistance

## Abstract

**Background:**

Glioblastoma is the most malignant tumor of the central nervous system and still lacks effective treatment. This study explores mutational biomarkers of 11 drugs targeting either the RTK/Ras/PI3K, the p53 or the Rb pathway using 25 patient-derived glioblastoma stem-like cell cultures (GSCs).

**Results:**

We found that *TP53* mutated GSCs were approximately 3.5 fold more sensitive to dual inhibition of mammalian target of rapamycin complex 1 and 2 (mTORC1/2) compared to wild type GSCs. We identified that Bcl-2(Thr56/Ser70) phosphorylation contributed to the resistance of *TP53* wild type GSCs against dual mTORC1/2 inhibition. The Bcl-2 inhibitor ABT-263 (navitoclax) increased sensitivity to the mTORC1/2 inhibitor AZD8055 in *TP53* wild type GSCs, while sensitivity to AZD8055 in *TP53* mutated GSCs remained unchanged.

**Conclusion:**

Our data suggest that Bcl-2 confers resistance to mTORC1/2 inhibitors in *TP53* wild type GSCs and that combined inhibition of both mTORC1/2 and Bcl-2 is worthwhile to explore further in *TP53* wild type glioblastomas, whereas in *TP53* mutated glioblastomas dual mTORC1/2 inhibitors should be explored.

## INTRODUCTION

Glioblastoma is the most frequently occurring primary malignant tumor of the central nervous system [1]. Since the introduction of temozolomide in 2005 [2], clinical studies testing novel agents for glioblastoma have failed to yield new therapeutic options [3, 4]. Given the heterogeneous mechanisms by which these drugs exert their anti-tumor activity, it is likely that only a subset of patients harboring specific molecular aberrations in their tumors would benefit from these agents. Therefore, it is imperative to preclinically identify predictive biomarkers that can be used to enrich clinical trials with patients who are likely to respond [5].

Recent studies have shown that glioblastomas contain aberrations largely in genes involved in Rb, p53 and receptor tyrosine kinase (RTK)/Ras/phosphatidylinositol-3-kinase (PI3K) signaling pathways [6, 7]. In order to discover therapeutic biomarkers that might aid in identifying glioblastoma patients most likely to respond to drugs targeting these pathways, we tested a panel of 11 different small molecule compounds on 25 patient-derived glioblastoma stem-like cell cultures (GSCs). GSCs are known to better preserve the gene expression profile [8], CNVs [8–10] and point mutations [9] of the parental tumor tissue than established cell lines. Furthermore, GSCs are more suitable for high-throughput drug screening compared to neurosphere cultures.

Herein, we present targeted exome sequencing of these 25 glioblastomas and drug sensitivity data of the corresponding patient-derived GSCs.

## RESULTS

### Exome sequencing data of parental glioblastoma tissue

To identify mutations present within our cohort of glioblastoma samples (N=25), we performed targeted exome sequencing of 1971 manually curated cancer-related genes. 16 patient samples were derived from primary glioblastomas, and the remaining 9 from relapsed glioblastomas. In total, 829 mutations were detected, ranging from 9-68 (average=21.5, SD=13.3) and 11-179 (average=53.9, SD=56.6) mutations in primary and relapsed glioblastomas, respectively. We identified 36 recurrently mutated genes (occurring three or more times) and 9 recurrent copy number variations (CNV) (Supplementary Table S1). *TTN, PTEN*, *EGFR* and *TP53* were amongst the top 4 most frequently mutated genes (Supplementary Table S1). *PTEN*, *EGFR* and *TP53* mutations have previously been implicated in gliomagenesis [7], whereas *TTN* most likely is a passenger mutation [11].

### Glioblastoma stem-like cell cultures respond heterogeneously to single compound treatments

To address the functional relevance of the 3 main deregulated pathways (RTK/Ras/PI3K, p53, Rb) in glioblastoma, we assembled a panel of 11 small molecule compounds either inhibiting the RTK/Ras/PI3K and Rb pathway, or reactivating the p53 pathway (Supplementary Table S2). We determined the GI50 (50% growth inhibitory concentration) after 8 days of drug exposure across 25 patient-derived GSCs. GSK2636771, a PI3KΔ-selective inhibitor, had a GI50 of >50 μM in several GSCs (data not shown), and was therefore excluded from further experiments due to its failure to inhibit cell proliferation potently.

**Figure 1:**
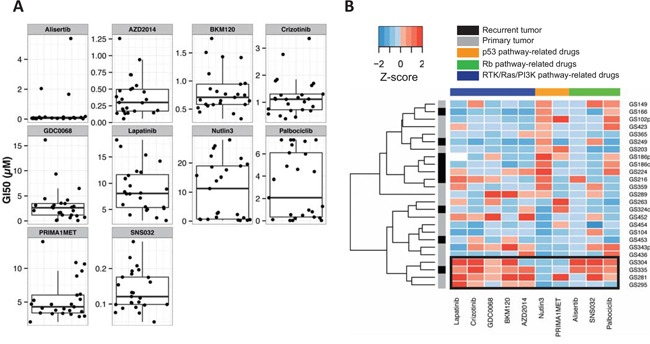
GI50 values of 25 GSCs for a panel of small molecule compounds **A.** Boxplot and dotplot in which each dot represents the GI50 value (μM) of a GSC to a specific compound. **B.** Supervised clustering of Z-transformed GI50 values (μM) was performed across the pathway-classified compounds. Unsupervised clustering was performed across the GSCs by complete linkage using euclidean distance. White, missing value; black rectangle, cluster of GSCs resistant to several compounds targeting the RTK/Ras/PI3K or Rb pathway.

We observed heterogeneous drug responses across the GSCs for 9 out of the remaining 10 compounds (GI50_SD_ >0.29 μM); only SNS-032 (CDK2/7/9 inhibitor) (GI50_average_ =0.14 μM, GI50_SD_ =0.056 μM) elicited a relatively homogeneous response across the 25 GSCs (Figure 1A). Unsupervised hierarchical clustering of Z-transformed drug sensitivity data did not reveal an obvious clustering pattern of the pathway-classified compounds (Supplementary Figure S1). Supervised clustering according to the pathway-classified compounds revealed a group of GSCs (4/25) which were on average at least 1.7 fold more resistant to 4 out of 5 RTK/Ras/PI3K targeting drugs and at least 1.5 fold more resistant to all (3/3) of the Rb pathway targeting compounds (Figure 1B). In contrast, this group was 5.7 fold more sensitive to the MDM2 inhibitor, Nutlin-3. There were no differences in drug sensitivity between primary and relapsed samples.

### *TP53* mutated GSCs are uniformly sensitive to dual mTORC1/2 inhibition but not uniformly sensitive to mTORC1 inhibition

In order to identify mutational biomarkers for the compounds used in this screen, we integrated the targeted exome sequencing data with the drug sensitivity data. To this end, we compared the GI50 values between the mutated and wild type samples for every gene containing a genetic aberration. We identified point mutations that were significantly correlated with GI50 values (unadjusted *p*<0.05, Wilcoxon rank-sum test) for 8 out of 10 compounds (Table 1). However, we identified CNVs (*PRKY* and *TBL1Y* amplifications) that were significantly correlated with GI50 values (unadjusted *p*<0.05, Wilcoxon rank-sum test) for only one compound (PRIMA-1^MET^) (Supplementary Table S3).

**Table 1:** Significant associations between mutated genes and drug response

Pathway	Compound	Target	Mutation	P (<0.05)	FDR
**RTK/Ras/PI3K**	Lapatinib	EGFR, ERBB2	*FBXW11*	0.043	0.55
	AZD2014	mTORC1, mTORC2	*TP53*	0.001	0.03
			*TRRAP*	0.002	0.03
			*IGF2R*	0.010	0.10
			*MLL5*	0.011	0.10
			*FBXW11*	0.036	0.26
	GDC-0068	AKT1, AKT2, AKT3	*TRRAP*	0.006	0.22
			*IGF2R*	0.020	0.36
	BKM120	Class I PI3K isoforms	*FBXW11*	0.006	0.11
		(p110α, β, γ and δ)	*MLL5*	0.008	0.11
			*TP53BP1*	0.014	0.11
			*RNF213*	0.015	0.11
			*SPEG*	0.015	0.11
			*TP53*	0.043	0.26
	Crizotinib	MET, ALK	*-*	-	-
**p53**	Nutlin3	MDM2	*MTOR*	0.010	0.13
			*PIKFYVE*	0.014	0.13
			*TYRO3*	0.014	0.13
			*FBXW11*	0.020	0.13
			*USP24*	0.020	0.13
			*TP53*	0.021	0.13
			*EGFR*	0.041	0.21
	PRIMA-1MET	Mutant p53	*RNF213*	0.004	0.15
		reactivation	*LAMA4*	0.027	0.49
	Alisertib	AURKA	-	-	-
**Rb**	Palbociclib	CDK4, CDK6	*HDAC9*	0.017	0.36
			*TRPM6*	0.036	0.36
			*PIK3R1*	0.040	0.36
	SNS-032	CDK2, CDK7, CDK9	*-*	-	-

The compounds are grouped according to the pathway they interact with. The genes indicate that GSCs with and without the specific gene mutations vary significantly in GI50 value in response to the specific drug (unadjusted *p*<0.05, Wilcoxon rank-sum test). FDR, false discovery rate.

**Figure 2:**
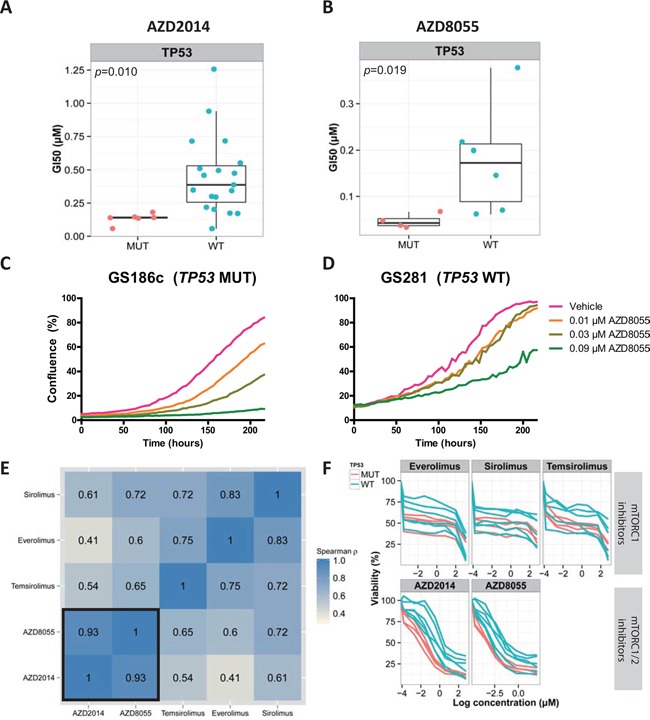
*TP53*^mut^ GSCs are uniformly sensitive to dual mTORC1/2 inhibition and not to mTORC1 inhibition **A, B.** Boxplot and dotplot in which each dot, stratified by their *TP53* mutation status, represents the GI50 values (μM) of AZD2014 or AZD8055 (dual mTORC1/2 inhibitors) for GSCs. **C, D.** Live-image monitoring of proliferation in response to increasing concentrations of AZD8055. **E.** Spearman correlation of the GI50 values (μM) of different mTORC1 and dual mTORC1/2 inhibitors for 10 GSCs. **F.** Dose-response curves of the same 10 GSCs. The colors indicate the *TP53* mutation status. Pink, *TP53*^mut^; blue, *TP53*^wt^; MUT, mutated; WT, wild type.

We found that *TP53* and *TRRAP* mutations were significantly associated with dual mTORC1/2 inhibition (FDR=0.026 and FDR=0.031, respectively, Wilcoxon rank-sum test) (Supplementary Figure S2). Sanger sequencing was used to validate the presence of the *TP53* mutations in the accompanying GSCs. Of the 6 *TP53* mutations identified by next-generation sequencing, all mutations were validated (6/6). GSCs with a *TP53* mutation (*TP53*^mut^) were more sensitive to the dual mTORC1/2 inhibitor AZD2014 than *TP53* wild type (*TP53*^wt^) GSCs (GI50_average_ =0.13 vs 0.45 μM, *p*=0.010, Wilcoxon rank-sum test). In contrast to the homogeneous response of the *TP53*^mut^ cultures (GI50_SD_=0.041 μM), *TP53*^wt^ cultures showed a more heterogeneous response (GI50_SD_=0.29 μM) (Figure 2A).

Next, we tested whether *TP53*^mut^ GSCs are also sensitive to the AZD2014 drug analogue, AZD8055. GI50 values of AZD2014 and AZD8055 were determined for 10 randomly selected GSCs after 5 days of drug exposure, and the results were highly correlated (*ρ*=0.93, spearman correlation) (Figure 2E). Similar to AZD2014 (GI50_*average*_ =0.22 versus 0.81 μM, *p*=0.010, Wilcoxon rank-sum test), *TP53*^mut^ GSCs were more sensitive to AZD8055 than *TP53*^wt^ GSCs (GI50_*average*_ =0.046 versus 0.18 μM, *p*=0.019, Wilcoxon rank-sum test) (Figure 2B). Live-cell imaging in response to 0.03 μM AZD8055 at t=200 hours confirmed that the *TP53*^mut^ GSC was inhibited more than the *TP53*^wt^ GSC (46% vs 7% inhibition) (Figure 2C, 2D).

To investigate whether *TP53*^mut^ GSCs were uniformly sensitive to classic rapamycin analogues (mTORC1 inhibitors), we tested the previous set of 10 GSCs for their response to sirolimus, temsirolimus and everolimus (Figure 2E). Although there was a moderate correlation between the GI50 values of mTORC1 inhibitors and dual mTORC1/2 inhibitors (*ρ* = 0.41-0.72, spearman correlation) (Figure 2E), there was no significant difference between *TP53*^mut^ and *TP53*^wt^ type GSCs in response to mTORC1 inhibitors (sirolimus, *p*=0.32; temsirolimus, *p*=0.26; everolimus, *p*=0.67, Wilcoxon rank-sum test). While there was an initial response to the rapamycin analogues at low doses, a steady state was reached at approximately 50% cell viability (Figure 2F). For AZD2014 and AZD8055 however, a stronger initial response was observed, and the steady state was reached at around 25% cell viability (Figure 2F).

### Phosphoproteome profiling identifies a Bcl-2 inhibitor as a drug combination partner for AZD8055

**Figure 3:**
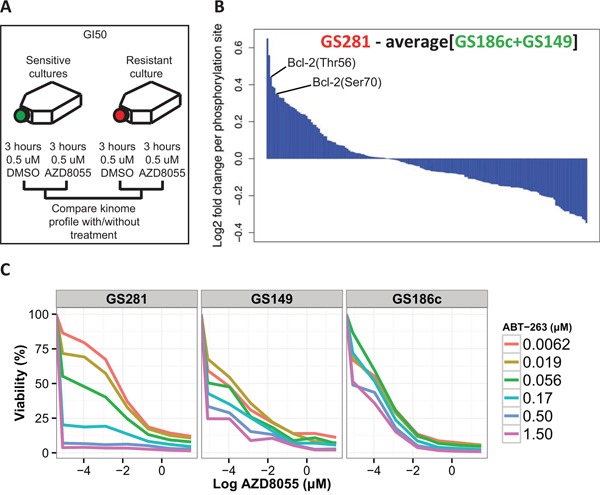
Kinome profiling identifies potential phosphosites implicated in resistance to AZD8055 **A.** Schematic depiction of the approach for dynamic kinome profiling. **B.** Waterfall plot of the 180 phosphosites. The values above and below 0 indicate respectively hyper- and hypophosphorylated phosphosites in the resistant *TP53*^wt^ (red) GSC relative to the sensitive *TP53*^mut^ (green) GSCs after exposure to AZD8055. **C.** Increasing doses of ABT-263 and AZD8055 were tested in the AZD8055-resistant *TP53*^wt^ GSC (GS281) and two AZD8055-sensitive *TP53*^mut^ GSCs (GS149 and GS186c).

The phosphoproteome is an important mediator of drug resistance. We investigated the phosphorylation pattern of 180 disease-relevant phosphosites using reverse phase protein arrays (RPPA) to identify possible therapeutic targets that might counter the resistance observed in *TP53*^wt^ GSCs. A resistant *TP53*^wt^ GSC (GS281) and 2 sensitive *TP53*^mut^ GSCs (GS149 and GS186c) were exposed to 0.5 μM AZD8055 or 0.5 μM vehicle (DMSO) for 3 hours (Figure 3A). Ribosomal protein S6(Ser235-236) and Akt(Ser473) phosphorylation (used as a measure of mTORC1 and mTORC2 activity, respectively [12]) decreased in the AZD8055-treated GSCs compared to vehicle-treated GSCs (data not shown), strongly indicating the on-target inhibitory effect of AZD8055 on mTORC1 and mTORC2.

We compared the RPPA profiles of sensitive *TP53*^mut^ GSCs (GS149 and GS186c) to the resistant *TP53*^wt^ GSC (GS281). We identified phosphosites that were specifically hyperphosphorylated in the AZD8055-resistant GSC relative to both AZD8055-senstive GSCs (Figure 3B). NDRG1(Thr346), Akt (Ser473), AMPKb1(Ser108), 4E-BP1(Thr70/Thr37.46), Bcl-2 (Thr56/Ser70), HSP27(Ser82), Cofilin(Ser3) were among the top hyperphosphorylated phosphosites in the AZD8055-resistant GSC relative to the AZD8055-sensitive GSCs.

Bcl-2 is a well known pro-survival protein broadly implicated in therapy resistance [13]. We combined the potent Bcl-2 inhibitor, ABT-263 [14], with AZD8055 to test for the reversal of resistance to AZD8055. Indeed, the addition of ABT-263 inhibited the proliferation of AZD8055-resistant GSC (GS281) more potently over a range of doses compared to AZD8055-sensitive GSCs (GS149 and GS186c) (Figure 3C).

## DISCUSSION

Targeted therapy has not been proven effective yet for the treatment of glioblastoma [3, 4]. An underlying reason for this failure may be due to the large intertumor heterogeneity of glioblastomas, as observed in sequencing studies [6, 7], rendering only a subset of tumors sensitive to a particular drug. It is therefore necessary to stratify patients or treatments guided by relevant predictive biomarkers [5]. Additionally, intratumor heterogeneity likely plays a major role in resistance to therapy [15]. Recent reports indicate that mutations can occur heterogeneously within a single glioblastoma [16, 17], and different tumor subclones can exhibit varying drug sensitivities [18, 19].

In this study, we focused on intertumor heterogeneity by identifying biomarkers of response using a panel of 25 GSCs derived from different patients. GSCs are known to better preserve the gene expression profile [8], CNVs [8–10] and point mutations [9] of the parental tumor tissue than established cell lines. Furthermore GSCs are more suitable for high-throughput drug screening compared to neurosphere cultures.

Targeted exome sequencing integrated with our compound screen identified that *TP53*^mut^ GSCs were uniformly sensitive to dual mTORC1/2 inhibition, but not mTORC1 inhibition alone; *TP53*^wt^ GSCs were more heterogeneous in response to dual mTORC1/2 inhibition.

To the best of our knowledge, this is the first report identifying *TP53* mutations as a biomarker for response to dual mTORC1/2 inhibition in glioblastoma. In other cancers, mTORC1 inhibition by rapamycin has previously been linked to selectively induce apoptosis in *TP53^mut^* rhabdomyosarcoma cell cultures [20, 21]. After rapamycin exposure, wild type p53 induces p21^cip^ expression, which leads to a G1 phase cell cycle arrest [20, 22]. In contrast, *TP53^mut^* or deficient cells undergo cell cycle progression and subsequent apoptosis [20, 22]. In this study however, we could not find any significant difference in sensitivity to mTORC1 inhibition between *TP53*^mut^ and *TP53*^wt^ GSCs. mTORC2 is increasingly recognized as an important mediator of gliomagenesis [23] and chemoresistance [24]. Future work is required to investigate whether *TP53*^mut^ cells cannot survive without mTORC2 activity, and whether mTORC2 specific inhibition would selectively kill *TP53^mut^* cells. The future development of mTORC2 specific inhibitors is important, since they may have a large therapeutic window as mTORC2 appears to be redundant in normal tissue [25].

We sought to understand the difference in response between *TP53*^mut^ AZD8055-sensitive and *TP53*^wt^ AZD8055-resistant GSCs. Through comparison of their RPPA profiles, we identified Bcl-2 as being implicated in resistance to AZD8055. These findings were supported by the observation that the *TP53*^wt^ AZD8055-resistant GSC was more sensitive to combined exposure of AZD8055 and ABT-263 than *TP53*^mut^ GSCs. Although this combination therapy was effective in *TP53*^wt^ GSCs, it is still unclear whether this is also the case *in vivo*. There are multiple studies showing that mTORC1/2 inhibitors can cross the blood-brain barrier (BBB) and interfere with glioblastoma growth in preclinical orthotopic *in vivo* models [26, 27], whereas it is still unknown whether ABT-263 can efficiently penetrate the BBB. Interestingly, in concordance with our findings, another study also found this combination to be synergistic in a subset of GSCs [28]. Collectively, our findings suggest that RPPA can be systematically used to identify phosphoproteins involved in resistance to therapy.

The clinical relevance of dual mTORC1/2 inhibition by AZD8055 or AZD2014 is yet to be fully elucidated. In a recent study, AZD8055 was shown to synergize with temozolomide, leading to a 30% prolonged survival of orthotopic glioma xenografts compared to treatment with either drug alone [26]. *TP53* is mutated or deleted in around 30% of glioblastomas [7]; for these patients, *TP53* mutations may be used as a biomarker to stratify patients for dual mTORC1/2 inhibitor treatment, thus enhancing the effect of combination therapy employing AZD2014 or AZD8055 with temozolomide.

In conclusion, our data suggest that *TP53* mutations are a predictive biomarker of response to dual mTORC1/2 inhibitors, rendering it worthwhile to further explore dual mTORC1/2 inhibitors in *TP53^mut^* glioblastomas. Furthermore, *TP53*^wt^ GSCs are resistant against mTORC1/2 inhibitors via Bcl-2; combined blockade of mTORC1/2 and Bcl-2 is worth exploring further in *TP53*^wt^ glioblastomas, or in *TP53^mut^* tumors to prevent the outgrowth of resistant subclones during mTORC1/2 inhibition.

## MATERIALS AND METHODS

### Patient-derived glioblastoma cultures

Resected human glioblastoma tumor material was obtained at the departments of neurosurgery of the Erasmus Medical Center (Rotterdam, The Netherlands) and Elisabeth Hospital (Tilburg, The Netherlands). Tumor material, that was safely accessible, was resected and used for research with the patient's written consent and in accordance with protocols approved by the institutional review board of the Erasmus Medical Center. A portion of the tumor tissue specimens was dissociated and maintained as adherent GSCs under serum-free culture conditions as was described previously [10]. The adjacent portion of the tumor tissue was snap-frozen and used for next-generation sequencing. 25 GSCs were used for the experiments.

### Next generation sequencing

We performed targeted exome sequencing of a “cancer mini-genome” consisting of 1971 cancer-related genes based on [29]. DNA was extracted using NorDiag Arrow (Isogen Life Science, De Meern, the Netherlands) and quantified with a Qubit 2.0 fluorometer (Life Technologies, Carlsbad, USA). Barcoded fragment libraries were generated from 600 ng of isolated DNA from tumor samples as previously described [30]. Pools of libraries were enriched for the cancer mini-genome using SureSelect technology. The enriched libraries were sequenced on a SOLiD 5500xl (Applied Biosystems, Foster City, USA) instrument according to the manufacturers’ protocol. Afterwards, the sequenced data were mapped to the reference genome (GRCh37/hg19) using Burrows-Wheeler Aligner [31]. Variant calling was performed using a custom pipeline as previously described [32]. Copy number status of a selected set of genes from the targeted sequencing data was estimated using modified Z-scores as described previously [33]. Copy number data from GS186p, GS289 and GS454 were excluded due to suboptimal quality.

### Small molecule compounds

The following small molecule compounds targeting the RTK/Ras/PI3K pathway were diluted in DMSO (Sigma-Aldrich, St. Louis, USA): lapatinib ditosylate was obtained from Santa Cruz Biotechnology (Dallas, USA) (catalog number 202205A). Crizotinib (S1068), NVP-BKM120 (S2247), GDC-0068 (S2808), AZD2014 (S2783), AZD8055 (S1555) and GSK2636771 (S8002) were obtained from Selleck Chemicals (Houston, USA).

The following small molecule compounds targeting the Rb pathway were diluted in DMSO: SNS-032 (S1145) was obtained from Selleck Chemicals. Palbociclib isethionate (PD-0332991) (S1579) was diluted in milliQ water and was obtained from Selleck Chemicals.

The following small molecule compounds targeting the p53 pathway were diluted in DMSO: Nutlin3 (S1061) and Alisertib (MLN8237) (S1133) were obtained from Selleck Chemicals. PRIMA-1MET (SC-361295) was obtained from Santa Cruz Biotechnology.

The following small molecule compound was identified as a potential combination partner of AZD8055 and was diluted in DMSO: ABT-263 (11500) was obtained from Cayman Chemical (Ann Arbor, USA).

### Viability assay

All viability assays were performed with GSCs below passage 20 using the CellTiter-Glo assay (Promega, WI, USA). Dose–response assays were performed in three step dilutions at six different concentrations in order to determine the growth inhibitory concentration required to inhibit 50% of cell proliferation (GI50). The 96-well plates were coated with matrigel (1:20, Trevigen, MD, USA) and seeded at 0.8-1.0×10^3^ cells/well and were left to adhere for 24 hours. After 24 hours the compounds were added to the wells. Cell viability was measured on day five or eight after treatment by using the luminescent CellTiter-Glo assay (Promega, WI, USA) according to the manufacturer's protocol. Luminescence was measured with a Tecan Infinite Reader (Tecan Group Ltd., Männedorf, Switzerland). The GI50 values were calculated by median effect equation [34].

### Live-cell imaging

The cells were seeded at 1.0×10^4^ cells/well in a matrigel coated (1:20) 24-well plate and were left to adhere for 24 hours. Hereafter DMSO (control) and AZD8055 were added at the indicated concentrations in duplicate. The 24-well plates were placed in an IncuCyte (Essen BioScience, MI, USA) at 37°C in a humid 95% air/5% CO2 chamber. Three phase contrast images/well were collected with a 10× objective every 4 hours for up to 9 days after addition of AZD8055. The percentage of the area of the well occupied by cells (confluence) was calculated using software of the IncuCyte live-cell imaging system (Essen BioScience).

### Reverse phase protein array (RPPA)

One AZD8055-resistant *TP53*^wt^ GSC (GS281) and 2 AZD8055-sensitive *TP53*^mut^ GSCs (GS149 and GS186c) were analyzed by RPPA. First, the GSCs were cultured until 80% confluence in matrigel coated (1:20, Trivigen) T175 flasks. Afterwards the cells were trypsinized and transferred to non-coated T175 flasks where they were cultured for 24 hours as neurospheres. Then, cells were exposed to 0.5 μM AZD8055 for 3 hours. Hereafter cell pellets were prepared following Carna Biosciences’ protocol and stored at −80°C. The cell pellet was thawed on ice and cell lysates were prepared in 50 μl lysis buffer following Carna Biosciences’ protocol. The protein concentrations were measured using the Bradford protein assay and were provided to Carna Biosciences together with the cell lysates. The lysates were further processed and analyzed by Carna Biosciences (Kobe, Japan) according to their protocol. In short, the lysates were spotted onto glass slides and immunostaining was performed with 180 antibodies (one antibody per slide). The signals were then measured as fluorescence of the fluorophore-labeled secondary antibodies and normalized with the values for gamma-tubulin (loading control). The raw data and calculated relative concentrations were reported back. For each GSC the AZD8055-treated condition was divided by the DMSO-treated values. As a result, the fold change between the AZD8055-treated and DMSO-treated conditions was derived for each phosphosite of the 3 GSCs. The fold change of the AZD8055-resistant *TP53*^wt^ GSC (GS281) were divided by the averaged fold change of the 2 AZD8055-sensitive *TP53*^mut^ GSCs (GS149 and GS186c). This enabled the identification of phosphosites that were hyperphosphorylated in the AZD8055-resistant GSC (GS281) compared to the 2 AZD8055-sensitive GSCs (GS149 and GS186c).

### Analysis of mutations in relation to drug sensitivity

The growth inhibitory concentration required to inhibit 50% of cell proliferation compared to non-treated control cells (GI50) was correlated to the presence or absence of mutated genes. Wilcoxon rank-sum tests were performed to statistically compare the GI50 values of mutated and wild type cultures for each aberrant gene (mutated, amplified or deleted) recurring three or more times among the patient-derived glioblastoma cultures. The false discovery rate was used to correct for multiple testing.

## SUPPLEMENTARY FIGURES AND TABLES


