# Supplementary material for: TP53 mutated glioblastoma stem-like cell cultures are sensitive to dual mTORC1/2 inhibition while resistance in TP53 wild type cultures can be overcome by combined inhibition of mTORC1/2 and Bcl-2
Source: Oncotarget. 2016 Aug 11;7(36):58435–44. doi: 10.18632/oncotarget.11205 (PMC5295441; doi:10.18632/oncotarget.11205)
Supplement: Supplementary file 1 [file oncotarget-07-58435-s001.pdf]

TP53 mutated glioblastoma stem-like cell cultures are sensitive to dual mTORC1/2 inhibition while resistance in *TP53* wild type cultures can be overcome by combined inhibition of mTORC1/2 and Bcl-2

SUPPLEMENTARY FIGURES AND TABLES

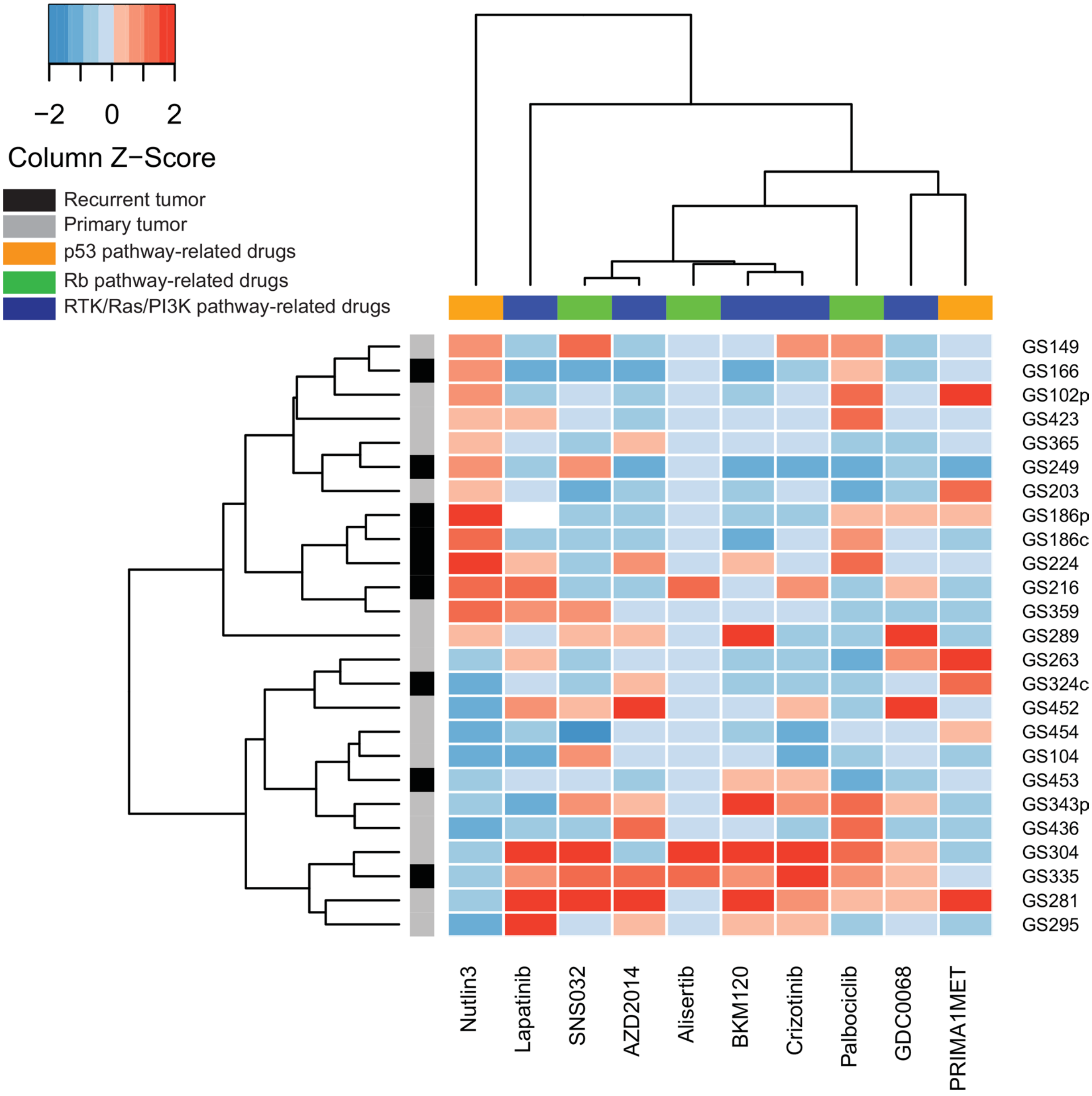

Supplementary Figure S1: Unsupervised clustering of Z-transformed GI50 values (µM) was performed by complete linkage using euclidean distance; white, missing values.

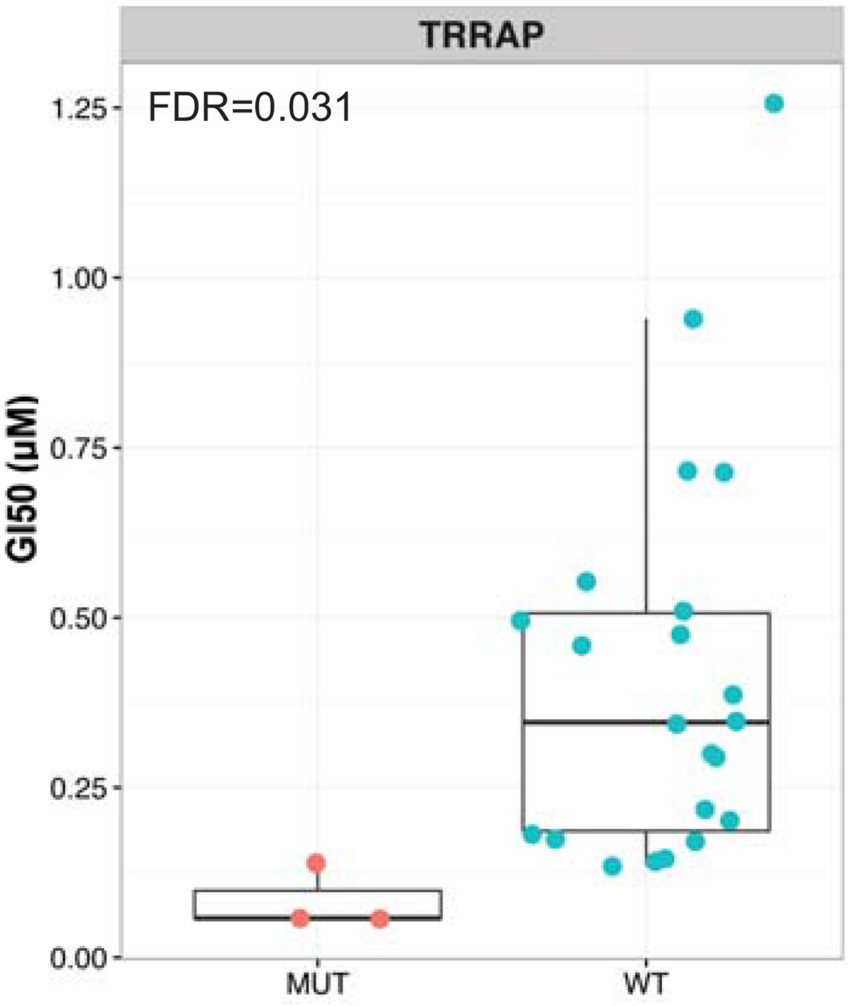

Supplementary Figure S2: *TRRAP*^{mut} GSCs appear sensitive to dual mTORC1/2 inhibition. Boxplot and dotplot in which each dot, stratified by its *TRRAP* mutation status, represents the GI50 value (µM) of AZD2014 (dual mTORC1/2 inhibitor) for a specific GSC.

Supplementary Table S1: Recurrent gene aberrations in the cohort of 25 glioblastomas

Gene	Number of recurring point mutations	Number of recurring CNVs
TTN	13	0
PTEN	9	0
EGFR	7	7
TP53	6	0
OBSCN	5	0
LRP2	4	0
MLL5	4	0
RNF213	4	0
SETD2	4	0
SPEG	4	0
AIM1	3	0
CUBN	3	0
FAT3	3	0
FBXW11	3	0
HDAC9	3	0
IGF2R	3	0
KDM3B	3	0
LAMA4	3	0
MAST2	3	0
MLL2	3	0
MSH6	3	0
MTOR	3	0
NF1	3	0
PIK3R1	3	0
PIKFYVE	3	0
PLCG1	3	0
PTPRD	3	0
RPGR	3	0
TAF1	3	0
TNFRSF10A	3	0
TP53BP1	3	0
TRPM6	3	0
TRRAP	3	0
TYRO3	3	0
UBR5	3	0
USP24	3	0
ERCC4	0	3
KDM5D	0	9
PRKY	0	5
RASGRP1	0	3
RPS6KA5	0	3
SIX4	0	3
TBL1Y	0	5
UTY	0	10

These genes were mutated, amplified or deleted three or more times in the cohort of parental tissues from which the 25 GSCs were derived. CNV, copy number variation.

Supplementary Table S2: Panel of small molecule compounds used in the initial drug screen on the 25 GSCs

Pathway	Compound	Target
**RTK/Ras/PI3K**	Lapatinib	EGFR, ERBB2
	AZD2014	mTORC1, mTORC2
	GDC-0068	AKT1, AKT2, AKT3
	BKM120	Class I PI3K isoforms (p110α, β, γ and δ)
	GSK2636771	PI3Kβ-Selective
	Crizotinib	MET, ALK
**p53**	Nutlin-3	MDM2
	PRIMA-1MET/APR-246	Mutant p53 reactivation
	Alisertib (MLN8237)	AURKA
**Rb**	PD0332991	CDK4, CDK6
	SNS-032	CDK2,7, 9

The compounds are grouped according to the pathway they interact with. GSK2636771 was excluded during the drug screen due to non-responsiveness of the GSCs.

Supplementary Table S3: Significant associations between CNVs and drug response

Pathway	Compound	Target	CNV	P (<0.05)	FDR
**RTK/Ras/PI3K**	Lapatinib	EGFR, ERBB2	-	-	-
	AZD2014	mTORC1,mTORC2	-	-	-
			-	-	-
			-	-	-
			-	-	-
			-	-	-
	GDC-0068	AKT1, AKT2, AKT3	-	-	-
			-	-	-
	BKM120	Class I PI3K isoforms	-	-	-
		(p110α, β, γ and δ)	-	-	-
			-	-	-
			-	-	-
			-	-	-
			-	-	-
	Crizotinib	MET, ALK	-	-	-
**p53**	Nutlin3	MDM2	-	-	-
			-	-	-
			-	-	-
			-	-	-
			-	-	-
			-	-	-
			-	-	-
	PRIMA-1MET	Mutant p53	*PRKY* amplification	0.033	0.15
		reactivation	*TBL1Y* amplification	0.033	0.15
	Alisertib		-	-	-
**Rb**	Palbociclib	CDK4, CDK6	-	-	-
			-	-	-
			-	-	-
	SNS-032	CDK2, CDK7, CDK9	-	-	-

The compounds are grouped according to the pathway they interact with. The genes indicate that GSCs with and without the specific gene CNVs vary significantly in GI50 value in response to the specific drug (unadjusted $p<0.05$, Wilcoxon rank-sum test). CNV, copy number variation; FDR, false discovery rate.
